# LINC00689 promotes prostate cancer progression via regulating miR-496/CTNNB1 to activate Wnt pathway

**DOI:** 10.1186/s12935-020-01280-1

**Published:** 2020-06-05

**Authors:** Liwei Meng, Zhonghai Li, Ye Chen, Deqian Liu, Zhaoxu Liu

**Affiliations:** 1 Department of Urology, Qilu Hospital of Shandong University, No.107 Culture Road, Jinan, 250000 Shandong China; 2 Department of Urology, Affiliated Hospital of Jining Medical University, Jining, 272000 Shandong China; 3 School of Nursing, Shandong University, No.44 Culture Road, Jinan, 250000 Shandong China

**Keywords:** Prostate cancer, LINC00689, miR-496, CTNNB1, Wnt pathway

## Abstract

**Background:**

Accumulating evidence has proved the significant influence of long non-coding RNAs (lncRNAs) in cancer formation and development, including PCa.

**Methods:**

The role of LINC00689 in PCa was confirmed by RT-qPCR, MTT, colony formation, flow cytometry, western blot and transwell assays. Besides, the binding ability between LINC00689 and miR-496 was validated by using luciferase reporter assay. Then RT-qPCR, RIP and luciferase reporter and western blot assays were employed to verify the interactions among LINC00689, miR-496 and CTNNB1. Furthermore, the rescuing role of CTNNB1 in Wnt pathway was proved by RT-qPCR, TOP/FOP Flash and western blot assays.

**Results:**

LINC00689 was upregulated in PCa tissues and cells as well as at the terminal stage. Further, knock down of LINC00689 repressed PCa cell proliferation, migration and invasion, and initiated PCa cell apoptosis. Additionally, miR-496 inhibitor and pcDNA3.1/CTNNB1 could neutralize the prohibitive effects of LINC00689 silencing on cell proliferation, migration and invasion, meanwhile, could offset the encouraging role of knocking down LINC00689 in cell apoptosis. Moreover, CTNNB1 upregulation exerted redemptive function in Wnt pathway inhibited by LINC00689 depletion.

**Conclusions:**

To sum up, LINC00689 promotes PCa progression via regulating miR-496/CTNNB1 to activate Wnt pathway, which may contribute to research about new targets for PCa treatment. 
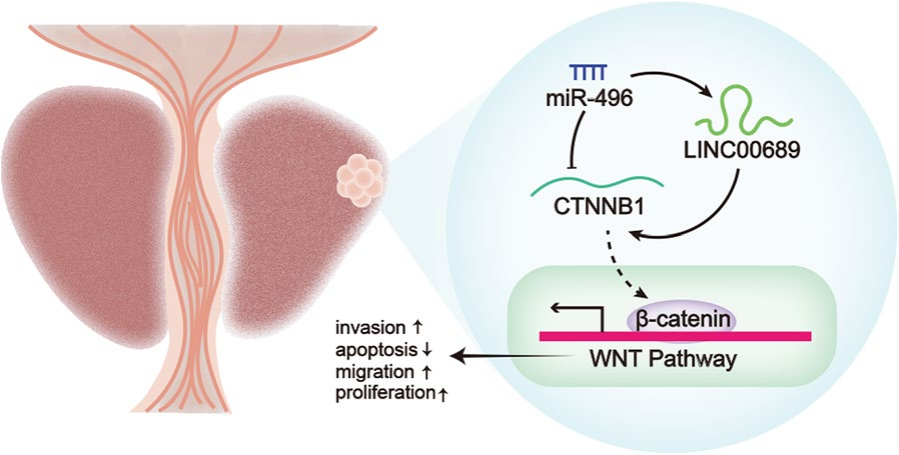

## Background

Prostate cancer (PCa) is identified as a type of the most common male malignancies in the world, with an increasing incidence and mortality in recent years [1–3]. The epidemiological survey shows that in the past 10 years, the developed degree of a country is negatively correlated with the death rate of PCa patients, that is, the more backward the country, the higher the fatality rate of PCa [4]. Considering the clinical value of PCa, the occurrence of tumors and effective treatment methods need to be studied in-depth.

Long non-coding RNAs (lncRNAs) were initially identified as the “garbage” of genomic transcription. Nevertheless, recent researches have elucidated that lncRNAs are involved in regulating molecular processes, such as X-chromosome silencing, gene imprinting, chromatin modification, transcriptional activation, transcriptional interference, and intra-nuclear transport, which begin to attract widespread attention [5–10]. During the development of PCa, lncRNAs play an important regulatory role. For instance, androgen-induced lncRNA SOCS2-AS1 facilitates PCa cell proliferation and prohibits apoptosis [11]. LncRNA MALAT-1 is recognized as a newly-found possible therapy target for PCa with castration resistance [12]. Low BDNF-AS expression is related to the unsatisfactory prognosis of PCa patients [13]. Further, LINC00689 has recently drawn attention when studying its role in cancer progression. However, the number of the concerned research is limited [14]. Therefore, the regulation mechanism of LINC00689 in PCa remains a novel topic of concern in this study.

In our research, LINC00689 promotes cell proliferation, migration, invasion as well as suppresses cell apoptosis via regulating miR-496/CTNNB1 to activate Wnt pathway, which may contribute to find a fresh target for PCa treatment.

## Methods

### Tissue samples

80 patients chosen from Affiliated Hospital of Jining Medical University were included in this research. None of the patients underwent chemo- or radiation therapy. Following surgical resection, tumor tissues were quickly frozen in liquid nitrogen and subsequently stored at − 80 °C for further use. The present research was favored by the Ethics Committee of Affiliated Hospital of Jining Medical University. Informed consent was attained from all the patients.

### Cell culture

Normal prostate epithelial cell (RWPE1) and PCa cells (DU145, LNCaP, PC-3 and C42B) were bought from American Type Culture Collection (ATCC; Manassas, VA, USA). Cells were cultured in line with previous description [15]. They were cultured with 10% FBS and 1% antibiotics in DMEM (Gibco, Rockville, MD, USA). In order to activate the Wnt/β-catenin signaling pathway, DU145 cells were treated with lithium chloride (LiCl; Sigma-Aldrich, St. Louis, MO, USA) for 24 h.

### Cell transfection

Specific shRNAs against LINC00689 (sh-LINC00689#1 and sh-LINC00689#2) and their corresponding NC (sh-NC), as well as the pcDNA3.1 vector containing the whole sequence of LINC00689 or CTNNB1 and the empty vector, were attained from Genechem (Shanghai, China). The miR-496 mimics, miR-496 inhibitors, NC mimics and NC inhibitors were constructed by GenePharma (Shanghai, China). By use of Lipofectamine 3000 (Invitrogen, Carlsbad, CA, USA), plasmids mentioned were individually transfected into DU145 or LNCaP cells in 24-well plates for 48 h. Sequences for shRNAs were listed as follows: sh-NC: CCGG TCTTGCGTCGTCTGTCTATAC CTCGAG GTATAGACAGACGACGCAAGA TTTTTG; sh-LINC00689#1: CCGG GCGTCTTTCCTTCTGTTAAGC CTCGAG GCTTAACAGAAGGAAAGACGC TTTTTG; CCGG GCTTCTGCTTTCCTGAAATTC CTCGAG GAATTTCAGGAAAGCAGAAGC TTTTTG. Plasmids’ sequences were shown as follows: NC mimics: gcugcauaucaguaucuacaug; miR-496 mimics: ugaguauuacauggccaaucuc; NC inhibitors: uagacaggcauguaauguacuc; miR-496 inhibitors: gagauuggccauguaauacuca.

### RT-qPCR (real-time quantitative polymerase chain reaction)

Total RNAs were extracted from tissues or cells by utilizing TRIzol reagents (Invitrogen), and then reverse-transcribed into cDNA in line with the protocol of a reverse transcriptase kit (Takara, Dalian, China). Next, RT-qPCR was undertaken with TB Green Premix ExTaq II (Takara) at the Applied Biosystems 7500 Real Time PCR system (Applied Biosystems, Foster City, CA, USA). Relative gene expression was normalized to GAPDH or U6. Quantification of relative gene expression was conducted via comparative 2^−∆∆Ct^ approach. Assay was undertaken in at least triplicate. Primer sequences were listed as follows: LINC00689 (F): AGTTGGTACAGGGAGGGGTT; LINC00689 (R): GTCCCTCTTGGTGGAGTTGG; miR-496 (R): tgagtattacatggccaatctc; miR-496 (F): GCCGAGtgagtattacatggcc; GAPDH (F): GGAGCGAGATCCCTCCAAAAT; GAPDH (R): GGCTGTTGTCATACTTCTCATGG; U6 (F): CTCGCTTCGGCAGCACA; U6 (R): AACGCTTCACGAATTTGCGT; CTNNB1 (F): ACGGAGGAAGGTCTGAGGAG; CTNNB1 (R): AGCCGCTTTTCTGTCTGGT.

### MTT (methyl thiazolyl tetrazolium) assay

Cell viability was explored with the MTT assay kit (Invitrogen). In short, transfected DU145 or LNCaP cells were seeded at the density of 1 × 10^3^ cells per well to 96-well plates, washed in phosphate-buffered saline (PBS; Sigma-Aldrich) and sequentially incubated for 4 h using MTT solution. Upon incubation, medium was exchanged into DMSO and cells were incubated for 10 min. OD490 nm value was read with a microplate reader. Assay was implemented in at least triplicate.

### Colony formation assay

Transfected DU145 or LNCaP cells were harvested after 48 h and added onto 6-well plates. 14 days later, cells were fixed by 4% formaldehyde (Sigma-Aldrich) for 30 min, stained for 5 min in 0.1% crystal violet solution (Sigma-Aldrich). Colonies were photographed by a camera (Canon, Japan) and the visible colonies containing over 50 cells were counted, manually. Assay was performed in at least triplicate.

### Cell apoptosis assay

The Annexin V-FITC Apoptosis Detection Kit (BD, San Diego, CA, USA) was employed to examine cell apoptosis. Transfected DU145 or LNCaP cells were washed in cold PBS, followed by re-suspended in 6-well plates with 200 μL binding buffer (Thermo Fisher Scientific, Waltham, MA, USA) adding fluorescein isothiocyanate (FITC)-labeled annexin V and PI (Sigma-Aldrich). Upon incubation without light for 30 min, 300 μL binding buffer was supplemented. Flow cytometry (Beckman Coulter, Brea, CA, USA) was conducted for analysis. Assay was conducted in at least triplicate.

### Caspase-3 activity assay

Transfected DU145 or LNCaP cells were lysed in lysis buffer (Invitrogen) and cell protein extracts were added in Ac-DEVD-AMC (Beyotime, Haimen, China) and sequentially incubated for 1 h. The caspase-3 activity kit was procured from Beyotime and used as required. Suspension was then placed in the influorescence spectrometer (Promega, Madison, WI, USA) so as to analyze influorescent intensity at 405 nm. This assay was conducted in at least triplicate.

### Transwell assay

On the one hand, transfected DU145 or LNCaP cells (1 × 10^5^) were added in the upper uncoated (for migration) or 0.5 mm of standard Matrigel (Cat#356234, BD)-coated (for invasion) transwell chambers (8 μm pore size; BD) with serum-free medium. On the other hand, culture medium with 10% fetal bovine serum (FBS; PAN-Biotech, Adenbach, Bagolia, Germany) was loaded to the lower wells. 24 h later, the non-migratory or non-invasive cells were wiped out. The filters were fixed, and stained by crystal violet solution. Five randomly picked fields were counted per chamber using the inverted microscope (4 × objective lens). This assay was performed in at least triplicate.

### Bioinformatics analysis

MiRNAs that can bind with LINC00689 were predicted by starBase v3.0 (http://starbase.sysu.edu.cn/). The downstream target mRNAs of miR-496 were predicted from starBase v3.0 (http://starbase.sysu.edu.cn/) with five program numbers.

### Luciferase reporter assay

LINC00689 or CTNNB1 3′UTR fragments covering wild-type and mutant miR-496 binding sites were inserted into the pmirGLO dual-luciferase plasmid (Promega), and pmirGLO-LINC00689-WT/Mut or pmirGLO-CTNNB1 3′UTR-WT/Mut was thus formed. The pmirGLO-LINC00689-WT/Mut was co-transfected into DU145 or LNCaP cells with miR-496 mimics or NC mimics. The pmirGLO-CTNNB1 3′UTR-WT/Mut was co-transfected into DU145 or LNCaP cells with miR-496 mimics or miR-496 mimics + pcDNA3.1/LINC00689 or NC mimics. For detecting luciferase activities, dual luciferase reporter assay system (Promega) was applied. This assay was undertaken in at least triplicate.

### RNA immunoprecipitation (RIP)

The RIP assay was implemented via a Magna RIP RNA Binding Protein Immunoprecipitation Kit (Bersinbio, Guangzhou, China). DU145 or LNCaP cells were lysed adopting RIP lysis buffer. Cell lysates were then divided into two equivalent parts for incubating with either anti-Ago2 antibody (ab32381, Abcam, Cambridge, MA, USA) or non-specific anti-IgG antibody (ab190475, Abcam). Magnetic beads (Invitrogen) were supplemented to cell lysates and incubation was continued for 1 h, after which were incubated with Proteinase K (Absin, Shanghai, China) for 1 h at 55 °C. Detection of the enriched RNA was subjected to RT-qPCR. Assay was undertaken in at least triplicate.

### Western blot

Transfected DU145 or LNCaP cells with or without LiCl treatment were lysed in RIPA buffer (Thermo Fisher Scientific) with additional blends of protease inhibitors (Roche, Mannheim, Germany). Lysate was collected and subjected to SDS-PAGE (Bio-Rad, Hercules, CA, USA), after which were transferred onto PVDF membranes (Bio-Rad). Upon incubation with Blocking One reagent (Nacalai Tesque, Kyoto, Japan), membranes were blotted by primary antibodies against CTNNB1 (SAB2701829, Sigma-Aldrich), β-catenin (ab32572, Abcam), CCND1 (SAB1405510, Sigma-Aldrich), CDK2 (ab32147, Abcam), c-MYC (ab32072, Abcam), and GAPDH (ab245356, Abcam) and then by HRP-labeled secondary antibodies. The signals were monitored after washing using the ECL Western blot kit (Thermo Fisher Scientific). Assay was undertaken in at least triplicate.

### TOP/FOP flash

DU145 cells were co-transfected with sh-LINC00689#1 or sh-LINC00689#1 + pcDNA3.1/CTNNB1 or sh-LINC00689#1 + LiCl or sh-NC and TOP Flash or FOP Flash plasmids (Upstate Biotechnology, Lake Placid, NY, USA). For detecting luciferase activities, dual luciferase reporter assay system (Promega) was applied. Assay was undertaken in at least triplicate.

### Statistical analysis

All assays were undertaken in triplicate. P < 0.05 was considered statistically significant. For analyzing the experimental data which were expressed as mean ± SD, SPSS 17.0 software (IBM, Armonk, NY, USA) was used. Student’s t-test was conducted for comparing two groups, and one-way ANOVA was for comparing multiple groups. Survival rate was assayed with Kaplan–Meier approach, and difference was analyzed by a log-rank test.

## Results

### LINC00689 is upregulated in prostate cancer tissues and cells

**Fig. 1:**
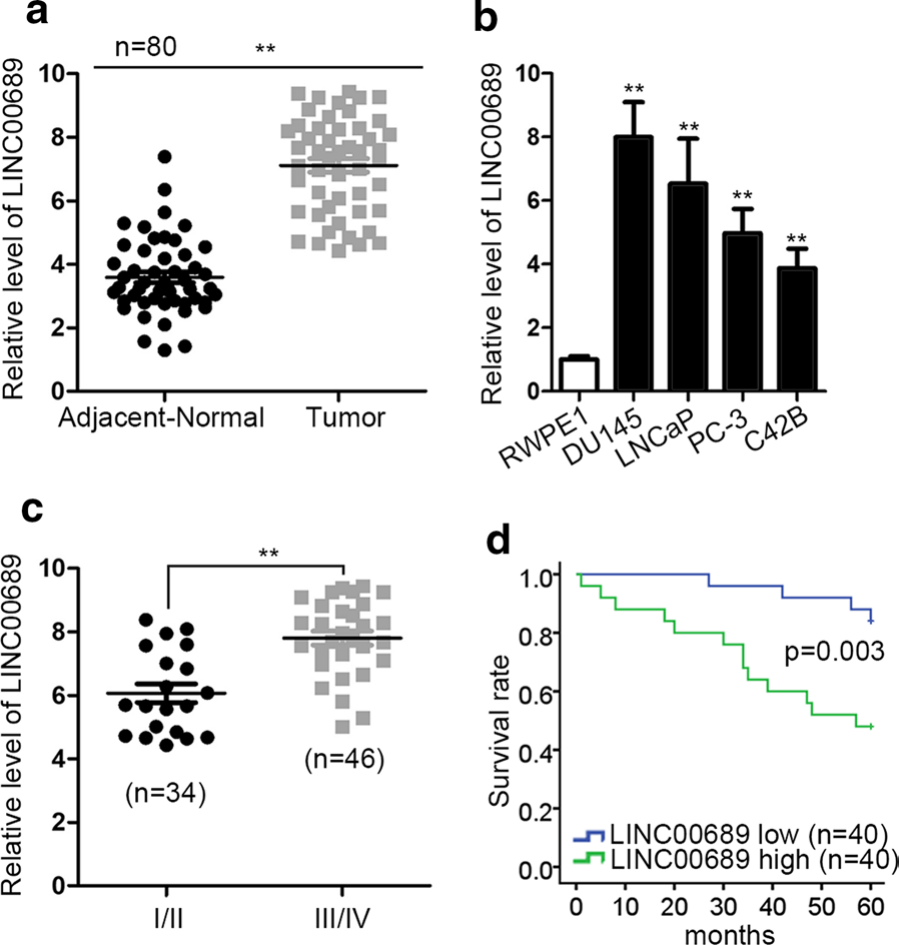
LINC00689 is upregulated in prostate cancer tissues and cells. **a** The expression of LINC00689 in adjacent-normal (n = 80) and tumor (n = 80) tissues was detected by RT-qPCR. **b** The expression of LINC00689 in normal prostate epithelial cell (RWPE1) and PCa cells (DU145, LNCaP, PC-3 and C42B) were detected by RT-qPCR. **c** Relative LINC00689 expression in different clinical stages (early I/II and advanced III/IV) was measured by RT-qPCR. **d** Kaplan–Meier curve and the log-rank test showed that the correlation between high or low LINC00689 expression and overall survival in patients with PCa. Error bars represent the mean ± SD of at least three independent experiments. ^**^P < 0.01

To verify the role of LINC00689 in PCa, RT-qPCR assessed that the expression of LINC00689 was much higher in PCa tissues than that in adjacent normal tissues (Fig. 1a). Meanwhile, LINC00689 expressed much higher in PCa cells (DU145, LNCaP, PC-3 and C42B) than control cells. Besides, DU145 and LNCaP contained the most expression of LINC00689 (Fig. 1b). Further, the patients with PCa at the advanced stage (III–IV) possessed much more LINC00689 expression than the patients at an early stage (I–II) (Fig. 1c). In addition, Kaplan–Meier curve in Fig. 1d depicted that high LINC00689 expression was closely associated with short overall survival time of PCa patients, indicating the unsatisfactory prognosis of PCa patients was induced by high LINC00689 level. Above data indicated that LINC00689 serves as a potential oncogene in PCa.

### Knock down of LINC00689 restrains prostate cancer progression

**Fig. 2:**
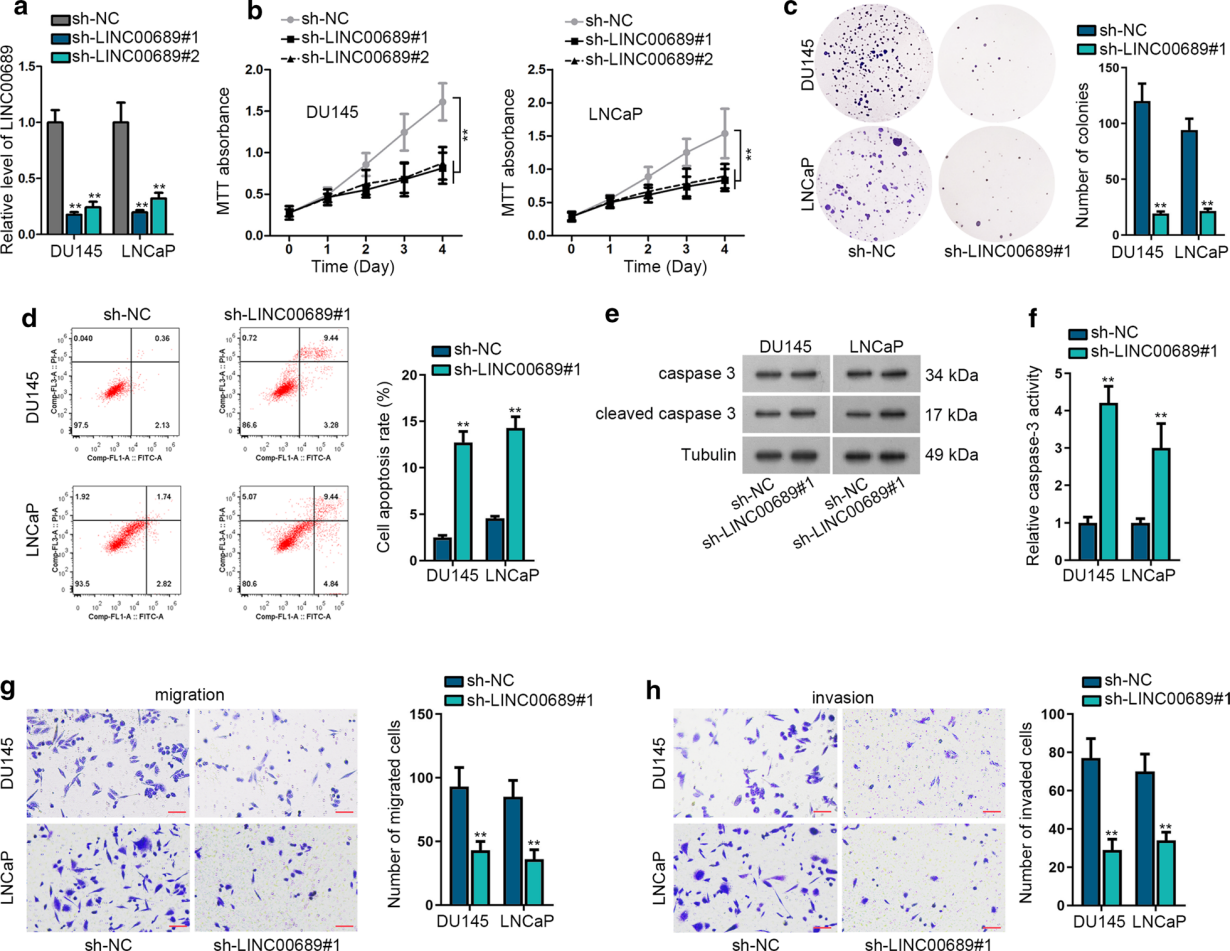
Knock down of LINC00689 restrains prostate cancer progression. **a** The knockdown efficiency of LINC00689 was detected in DU145 and LNCaP cells after 48 h of transfection with sh-LINC00689#1/#2. **b**, **c** MTT (detected after incubated for indicated time) and colony formation (measured after incubated for 14 days) assays measured the effects of knocking down LINC00689#1/#2 on DU145 and LNCaP cell proliferation. **d** Flow cytometry analysis measured the apoptosis in LINC00689 silenced DU145 and LNCaP cells. **e** Western blot assay examined that protein level of cleaved caspase 3 in sh-LINC00689#1 transfected DU145 and LNCaP cells. GAPDH was an internal control. **f** Caspase-3 test displayed the caspase-3 activity in response to LINC00689 downregulation in DU145 and LNCaP cells. **g**, **h** Transwell assay determined the effects of LINC00689 silencing on DU145 and LNCaP cell migration and invasion after incubated for 24 h. Images were captured by using the inverted microscope (4 × objective lens) (scale bar = 100 μm). Error bars represent the mean ± SD of at least three independent experiments. ^**^P < 0.01

For comprehending the regulation mechanism of LINC00689 in PCa, firstly, the knockdown efficiency of LINC00689 was detected in DU145 and LNCaP cells using sh-LINC00689#1/#2. The knockdown efficiency of sh-LINC00689#1 was better (Fig. 2a). Then followed by several functional assays, MTT assay validated that silencing of LINC00689 suppressed cell proliferation in DU145 and LNCaP cells. Hereinto, the effectiveness of LINC00689#1 was more evident (Fig. 2b). And colony formation assay confirmed the obstructive role of LINC00689 depletion in cell proliferation based on the decline in colony number of DU145 and LNCaP cells (Fig. 2c). With regard to cell apoptosis, flow cytometry detected that LINC00689 downregulation induced about two folds of increase in cell apoptosis rate in DU145 and LNCaP cells (Fig. 2d). Western blot assay measured the upregulated protein level of cleaved caspase 3 was caused by LINC00689 knockdown (Fig. 2e and Additional file 1a). Similarly, Caspase-3 test illustrated that absence of LINC00689 motivated cell apoptosis (Fig. 2f). Subsequently, transwell assay determined that shortage of LINC00689 prohibited cell migration and invasion referring with the reduced number of migrated and invaded cells (Fig. 2g, h). Above all, knockdown of LINC00689 restrains PCa progression.

### LINC00689 sponges with miR-496 in prostate cancer

**Fig. 3:**
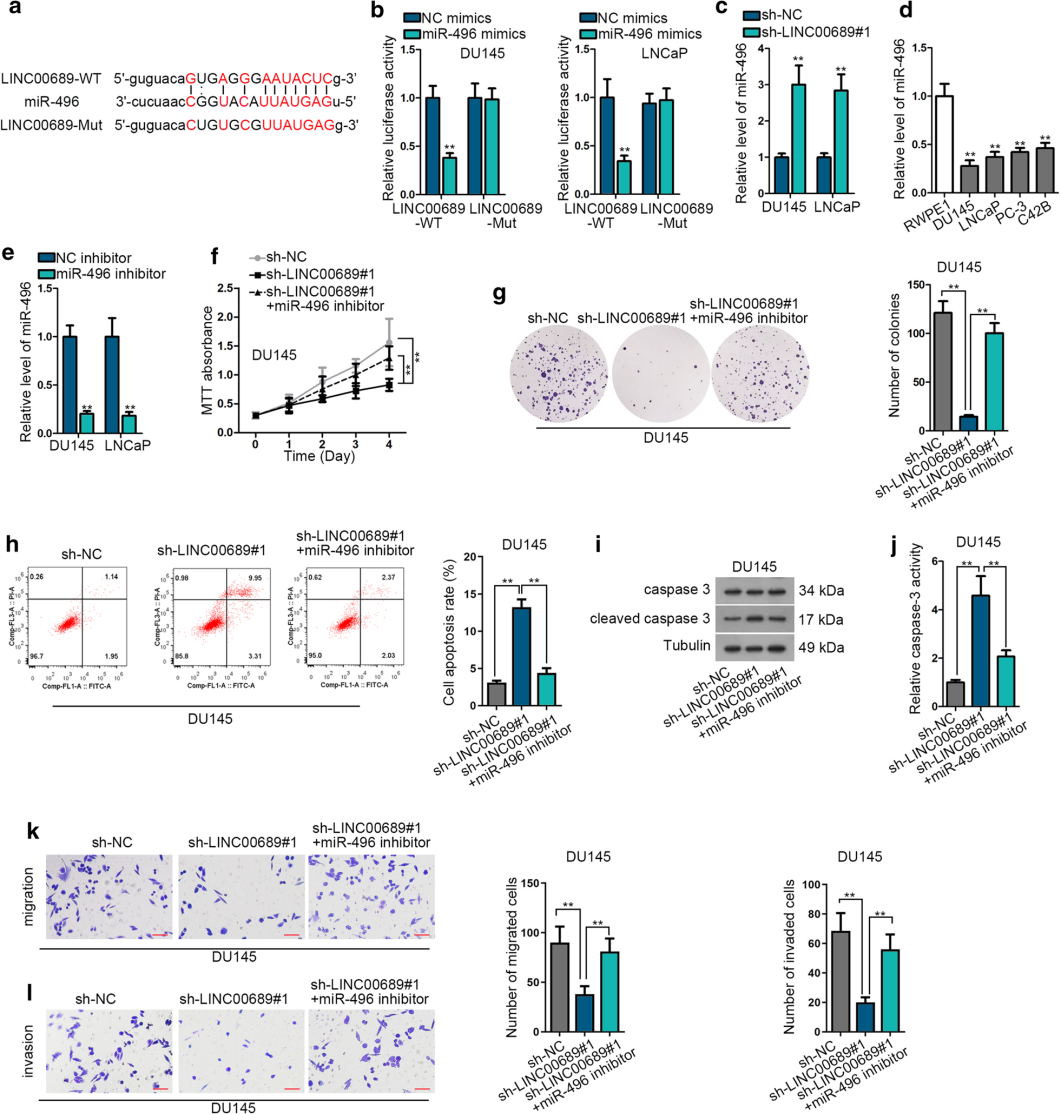
LINC00689 sponges with miR-496 in prostate cancer. **a** The binding sites of LINC00689-WT, miR-496 and LINC00689-Mut predicted by starbase were displayed. **b** Luciferase reporter assay examined the luciferase activity of LINC00689-WT/Mut in miR-496 mimics transfected DU145 and LNCaP cells. **c** RT-qPCR measured the expression of miR-496 in sh-LINC00689#1 transfected DU145 and LNCaP cells (post 48 h of transfection). **d** The expression of miR-496 in normal prostate epithelial cell (RWPE1) and PCa cells (DU145, LNCaP, PC-3 and C42B) was detected by RT-qPCR. **e** The knockdown efficiency of miR-496 was measured in miR-496 inhibitor transfected DU145 and LNCaP cells (48 h for plasmid transfection). **f**, **g** MTT (for indicated time) and colony formation (14 days later) assays detected DU145 cell proliferation in differently transfected groups (sh-NC, sh-LINC00689#1 and sh-LINC00689#1 + miR-496 inhibitor). **h** Flow cytometry analysis examined DU145 cell apoptosis in differently transfected groups (sh-NC, sh-LINC00689#1 and sh-LINC00689#1 + miR-496 inhibitor). **i** Western blot assay measured cleaved caspase 3 expression in differently transfected groups (sh-NC, sh-LINC00689#1 and sh-LINC00689#1 + miR-496 inhibitor). GAPDH was an internal control. **j** Caspase-3 test measured DU145 cell apoptosis in differently transfected groups (sh-NC, sh-LINC00689#1 and sh-LINC00689#1 + miR-496 inhibitor). **k**, **l** Transwell assay (24 h) measured DU145 cell migration and invasion in differently transfected groups (sh-NC, sh-LINC00689#1 and sh-LINC00689#1 + miR-496 inhibitor). Images were captured by using the inverted microscope (4 × objective lens) (scale bar = 100 μm). Error bars represent the mean ± SD of at least three independent experiments. ^**^P < 0.01

For the purpose of finding the microRNA (miRNA) sponged by LINC00689, we searched from starBase v3.0. Among all candidate miRNAs, miR-496 has been reported to be downregulated in PCa samples [16]. As shown in Fig. 3a and Additional file 2, the binding sites of LINC00689 and miR-496 predicted by starBase were displayed. Luciferase reporter assay confirmed the binding relation between LINC00689 and miR-496. Luciferase activity of LINC00689-WT was reduced in miR-496 mimics transfected cells (Fig. 3b). RT-qPCR measured that sh-LINC00689 increased the expression of miR-496 in DU145 and LNCaP cells (Fig. 3c). Moreover, the expression of miR-496 in normal prostate epithelial cell (RWPE1) and PCa cells (DU145, LNCaP, PC-3 and C42B) were detected, and miR-496 was downregulated in PCa cells in comparison with that in RWPE1 cell (Fig. 3d). The knockdown efficiency of miR-496 was also measured in miR-496 inhibitor transfected cells (Fig. 3e). Finally, the rescuing function of miR-496 inhibitor in sh-LINC00689 transfected cells was elucidated. MiR-496 inhibition canceled out restraining effects of sh-LINC00689 on cell proliferation (Fig. 3f, g). As for PCa cell apoptosis, in follow cytometry assay, when downregulating LINC00689, the DU145 cell apoptosis rate was three times as much as the control group. And cell apoptosis rate exhibited two folds of decrease in sh-LINC00689#1 + miR-496 inhibitor group compared with sh-LINC00689#1 group (Fig. 3h). In western blot analysis and caspase-3 activity detection, miR-496 inhibitor counteracted the encouraging effects of LINC00689 depletion on cleaved caspase 3 protein level and Caspase-3 activity (Fig. 3i, j and Additional file 1b). Similarly, the depressing function caused by LINC00689 insufficiency on cell migration and invasion was reversed by inhibition of miR-496 (Fig. 3k, l). In a word, miR-496 is sponged by LINC00689, and miR-496 inhibitor could partly rescue the suppressive role of LINC00689 knockdown in PCa cellular activities.

### MiR-496 targets to CTNNB1 in prostate cancer

**Fig. 4:**
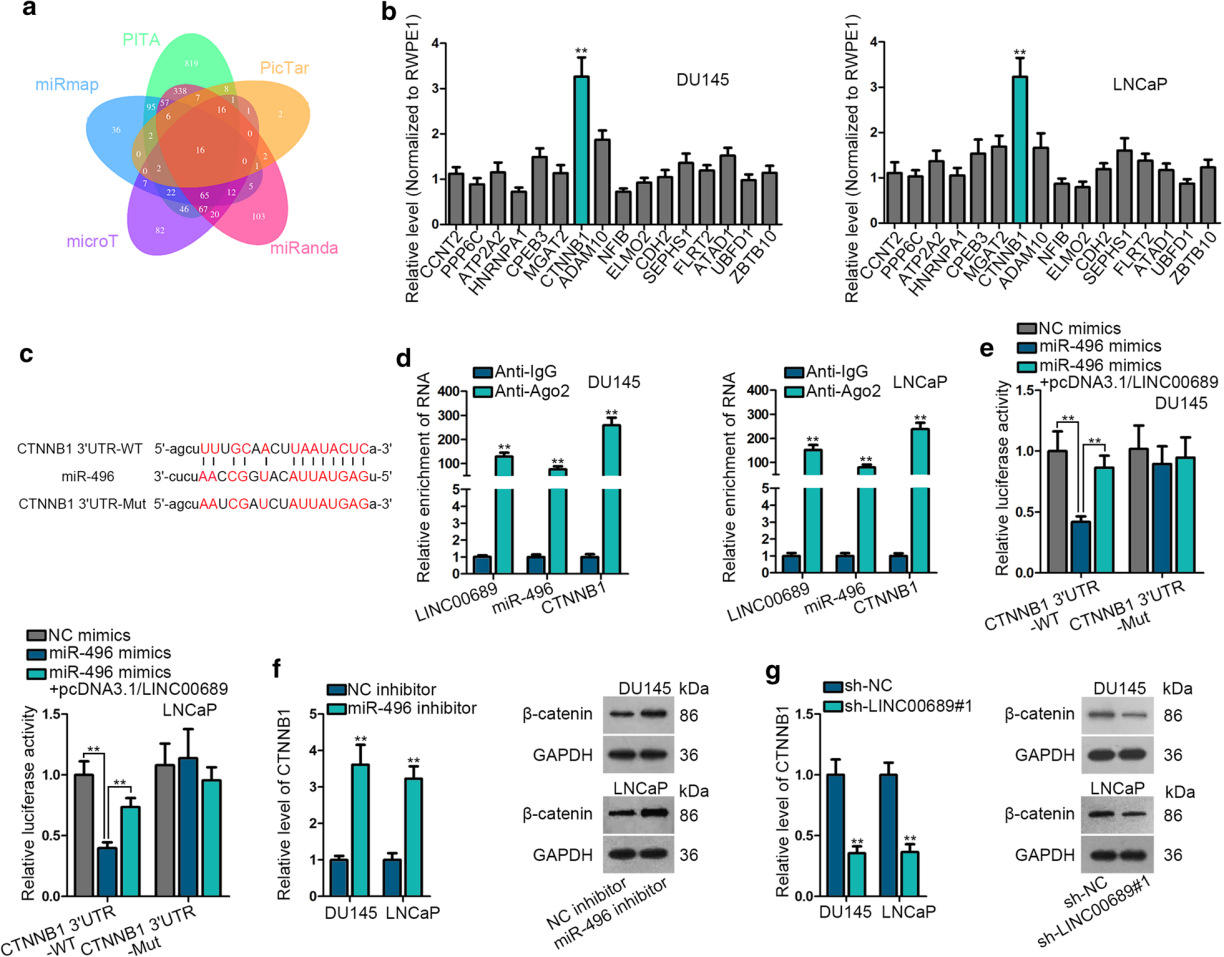
MiR-496 targets to CTNNB1 in prostate cancer. **a** Venn diagram exhibited 16 qualified messenger RNAs (mRNAs) collected by using starbase. **b** The expression of 16 mRNAs in normal prostate epithelial cell (RWPE1) and PCa cells (DU145 and LNCaP) was detected by RT-qPCR. **c** The conjectured binding sites between miR-496 and CTNNB1 were showed. **d** RIP assay detected the enrichment of LINC00689, miR-496 and CTNNB1 in Anti-IgG or Anti-Ago of DU145 and LNCaP cells. **e** Luciferase reporter assay examined the luciferase activity of CTNNB1 3′UTR-WT/Mut in transfected DU145 and LNCaP cells (NC mimics, miR-496 mimics and miR-496 mimics + pcDNA3.1/LINC00689). **f**, **g** RNA or protein level of CTNNB1 or β-catenin was measured respectively by RT-qPCR and western blotting in miR-496 inhibitor and sh-LINC00689#1 transfected DU145 and LNCaP cells (48 h for plasmid transfection). GAPDH was an internal control. Error bars represent the mean ± SD of at least three independent experiments. ^**^P < 0.01

Based on the above results, we needed to figure out the target gene of miR-496 to complete competing endogenous RNA (ceRNA) regulation mechanism of LINC00689 in PCa. By utilizing starBase, 16 qualified messenger RNAs (mRNAs) were plotted in Venn diagram (Fig. 4a). The expression of the indicated mRNAs in normal prostate epithelial cell (RWPE1) and PCa cells (DU145 and LNCaP) was detected by RT-qPCR. CTNNB1 was prominently upregulated in DU145 and LNCaP cells compared with normal prostate epithelial cell (RWPE1) (Fig. 4b). In hence, CTNNB1 was chose to perform the following assays. The predicted binding site between miR-496 and CTNNB1 was showed in Fig. 4c. And RIP assay validated that LINC00689, miR-496 and CTNNB1 coexisted in RISC (RNA-induced silencing complex) (Fig. 4d), indirectly confirming the interactions among these three genes in PCa cells. And luciferase reporter assay verified the binding facts between miR-496 and CTNNB1. Besides, pcDNA3.1/LINC00689 remedied the blocking effect of miR-496 mimics on luciferase activity of CTNNB1 3′UTR-WT (Fig. 4e), suggesting that LINC00689 could upregulate CTNNB1 via sponging miR-496. Moreover, the mRNA expression of CTNNB1 and protein level of β-catenin (protein form of CTNNB1) were measured in miR-496 inhibitor and sh-LINC00689 transfected cells, respectively. And obviously, the miR-496 inhibitor stimulated the mRNA and protein levels of CTNNB1, however, sh-LINC00689 led to completely opposite results (Fig. 4f, g). On the whole, CTNNB1 is targeted by miR-496, and the expression of CTNNB1 is negatively/positively regulated by miR-496/LINC00689 in PCa cells.

### LINC00689 activates Wnt pathway via upregulating CTNNB1

**Fig. 5:**
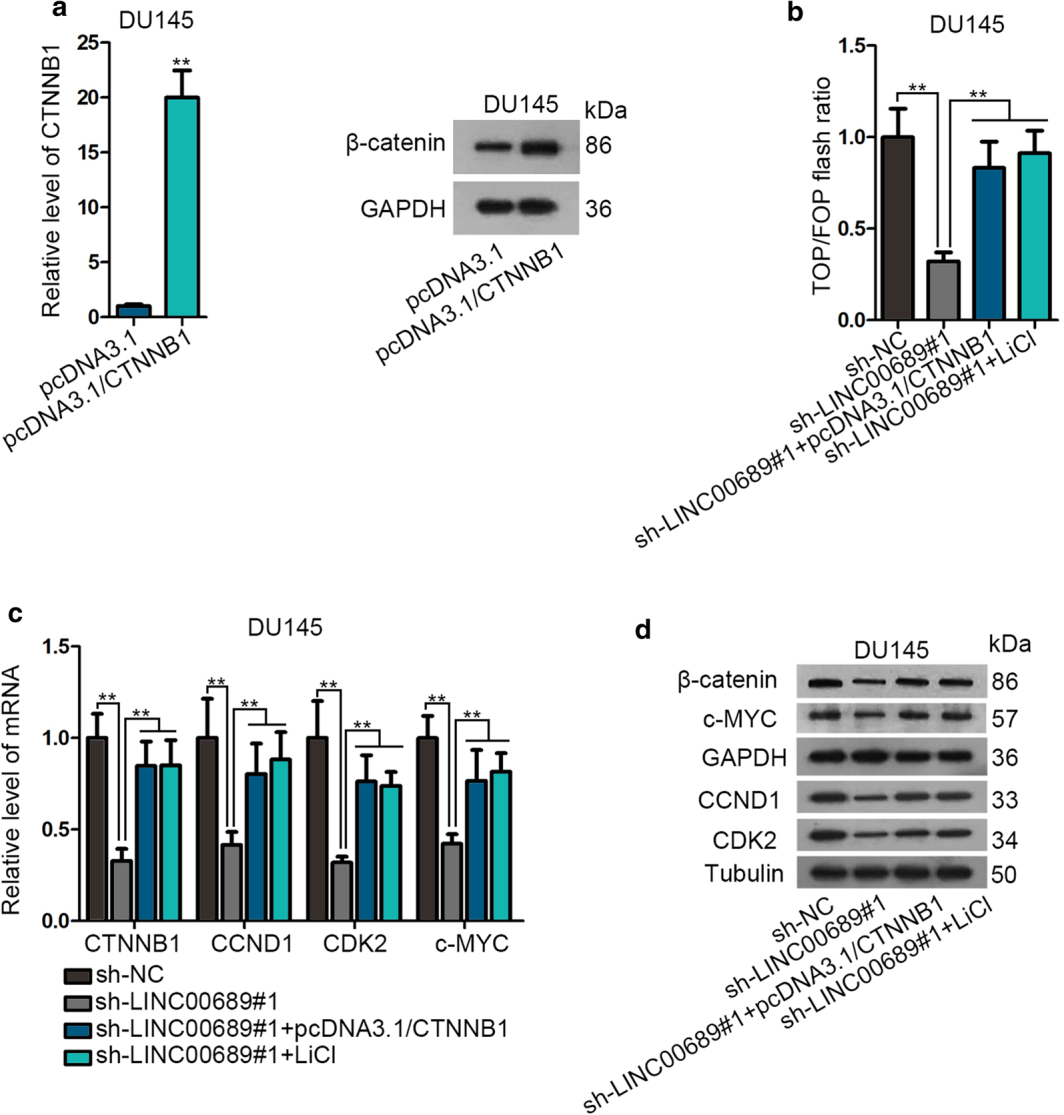
LINC00689 activates Wnt pathway via upregulating CTNNB1. **a** The overexpression efficiency of CTNNB1 was measured by RT-qPCR and western blotting in pcDNA3.1/CTNNB1 transfected DU145 cells (48 h for plasmid transfection). **b** TOP/FOP flash assay detected the luciferase activity of β-catenin in DU145 cells under differently transfected conditions (sh-NC, sh-LINC00689#1 and sh-LINC00689#1 + pcDNA3.1/CTNNB1 and sh-LINC00689#1 + LiCL) (24 h post LiCL treatment). **c**, **d** RT-qPCR and western blot assays detected the mRNA and protein levels of Wnt-related proteins (CTNNB1/β-catenin, CCND1, CDK2 and c-MYC) in DU145 cells under differently transfected conditions (sh-NC, sh-LINC00689#1 and sh-LINC00689#1 + pcDNA3.1/CTNNB1 and sh-LINC00689#1 + LiCL) (24 h post LiCL treatment). GAPGH was an internal control. Error bars represent the mean ± SD of at least three independent experiments. ^**^P < 0.01

Wnt pathway is a considerable element of researching cancer progression [17]. And the overexpression efficiency of CTNNB1 was measured by RT-qPCR and western blot assays in pcDNA3.1/CTNNB1 transfected DU145 cell (Fig. 5a). Then TOP/FOP flash assay suggested that pcDNA3.1/CTNNB1 or LiCl treatment offset the interceptive role of sh-LINC00689 in Wnt pathway. LINC00689 knockdown-mediated suppressed function on β-catenin activity was offset by CTNNB1 overexpression or LiCl treatment (Fig. 5b). RT-qPCR and western blot assays detected that mRNA and protein level of Wnt-related proteins (CTNNB1/β-catenin, CCND1, CDK2 and c-MYC) were reduced by knocking down LINC00689, then this inhibitory effect was counteracted by CTNNB1 upregulation or LiCl supplement (Fig. 5c, d). In short, LINC00689 activates Wnt pathway via upregulating CTNNB1 in PCa.

### LINC00689 promotes prostate cancer progression by upregulating CTNNB1

**Fig. 6:**
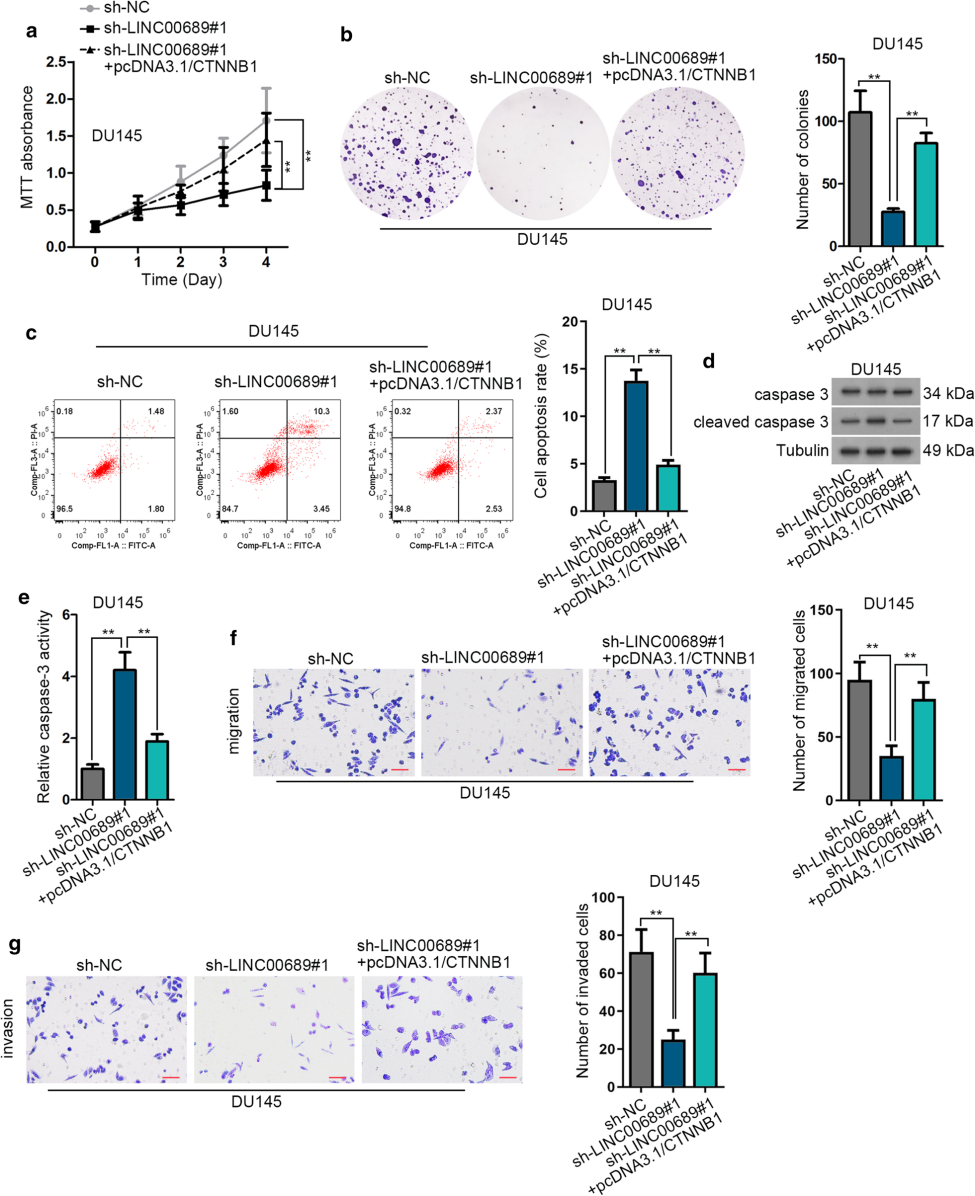
LINC00689 promotes prostate cancer progression by overexpressing CTNNB1. **a**, **b** MTT (cultured for 0, 1, 2, 3, 4 days) and colony formation (after 14 days of incubation) assays detected DU145 cell proliferation in differently transfected groups (sh-NC, sh-LINC00689#1 and sh-LINC00689#1 + pcDNA3.1/CTNNB1). **c** Flow cytometry measured DU145 cell apoptosis in differently transfected groups (sh-NC, sh-LINC00689#1 and sh-LINC00689#1 + pcDNA3.1/CTNNB1). **d** Western blot assay measured cleaved caspase 3 expression in differently transfected groups (sh-NC, sh-LINC00689#1 and sh-LINC00689#1 + pcDNA3.1/CTNNB1). GAPDH was an internal control. **e** Caspase 3 activity was measured in DU145 cell proliferation in differently transfected groups (sh-NC, sh-LINC00689#1 and sh-LINC00689#1 + pcDNA3.1/CTNNB1). **f**, **g** Transwell assay (incubated for 24 h) measured DU145 cell migration and invasion in differently transfected groups (sh-NC, sh-LINC00689#1 and sh-LINC00689#1 + pcDNA3.1/CTNNB1). Images were captured by using the inverted microscope (4 × objective lens) (scale bar = 100 μm). Error bars represent the mean ± SD of at least three independent experiments. ^**^P < 0.01

The rescuing role of miR-496 inhibitor in sh-LINC00689 transfected PCa cells has been validated, and whether LINC00689 regulated the development of PCa through CTNNB1 needed further investigation. Through MTT and colony formation assays, upregulation of CTNNB1 reversed the inhibiting effects of knocking down LINC00689 on cell proliferation (Fig. 6a, b). In a similar way, LINC00689 silencing spurred DU145 cell apoptosis, and this stimulating effect was partly offset by overexpressing CTNNB1. And for flow cytometry analysis results, in sh-LINC00689#1 group, cell apoptosis rate had about two folds of increase compared with sh-NC group. In sh-LINC00689#1 + pcDNA3.1/CTNNB1 group, cell apoptosis rate declined and decreased about two folds in comparison with sh-LINC00689#1 group (Fig. 6c). Additionally, CTNNB1 overexpression neutralized the activating influence of LINC00689 downregulation on cleaved caspase 3 protein expression and caspase 3 activity (Fig. 6d, e and Additional file 1c). On the other hand, transwell assay proved the rescuing role of pcDNA3.1/CTNNB1 in DU145 cell migration and invasion restrained by LINC00689 insufficiency (Fig. 6f, g). In general, LINC00689 promotes PCa progression by upregulating CTNNB1 expression Additional file 1, 2.

## Discussion

PCa is one of the most prevalent malignancies for men [18, 19]. Additionally, the older man is, and the more likely he is to suffer from PCa, and 70–80 years old is the peak of having PCa [20]. Nonetheless, family hereditary PCa occurs when men are relatively young, and nearly half of patients with family hereditary PCa are under 56 [21]. The therapeutic methods of PCa include chemotherapy, radiation treatment and operation. Currently, proton therapy has no damage to non-tumor tissue and is a safe and effective way for treating PCa [22]. Nevertheless, the exact etiology and pathogenesis of PCa are poorly comprehended. Therefore, efforts are needed to explore more effective and specific measures to prevent PCa.

And previous studies have confirmed the role of lncRNAs in the initiation and development of diverse cancers, such as gastric cancer [23, 24], hepatocellular carcinoma [25, 26], renal carcinoma [27, 28], breast cancer [29, 30]. Regarding PCa, there have been some related studies about the role of lncRNAs [31–33]. Nonetheless, the function of LINC00689 in PCa had not been illustrated yet. In our research, LINC00689 exhibited extremely high expression in PCa tissues and cells compared with control groups. Further, the expression of LINC00689 in patients with advanced PCa was higher than that in patients with early PCa. Meanwhile, the higher the expression of LINC00689, the shorter the overall survival time of patients with PCa. Subsequently, depletion of LINC00689 inhibited PCa cell proliferation, migration and invasion, but promoted PCa cell apoptosis, indicating that LINC00689 served as an oncogene in PCa.

LncRNAs usually sponge with microRNAs (miRNAs) in cancers [34–36]. In this study, LINC00689 could bind with miR-496, and LINC00689 negatively modulated the expression of miR-496. In addition, the tumor suppressor role of miR-496 has been illustrated in non-small cell lung cancer [37], lung adenocarcinoma [38], bladder cancer [39], and osteosarcoma [40]. In the present study, miR-496 suppression could neutralize the inhibitory effects of LINC00689 silencing on PCa cell progression, suggesting the anti-tumor function of miR-496 in PCa. In other words, LINC00689 could promote the development of PCa via inhibiting miR-496.

Messenger RNAs (mRNAs) are indispensable members of ceRNA together with lncRNAs and miRNAs in cancer progression [41–43]. In our study, CTNNB1 acted as a target gene of miR-496, and the expression of CTNNB1 was separately negatively and positively regulated by miR-496 and LINC00689 in PCa. Furthermore, LINC00689 could regulate CTNNB1 via sponging miR-496. In addition, Wnt is identified as an important pathway to regulate cell growth, apoptosis, migration and invasion [44–46]. In this study, LINC00689 knockdown inhibited Wnt pathway, and CTNNB1 upregulation rescued this restraining effect. In addition, overexpressing CTNNB1 counteracted the prohibitive impacts of LINC00689 insufficiency on cell proliferation, migration and invasion as well as the stimulating function of that on cell apoptosis.

## Conclusion

In a summary, LINC00689 promotes PCa progression via regulating miR-496/CTNNB1 to activate Wnt pathway, which possibly bring about new idea for PCa treatment.

## Supplementary information

**Additional file 1.** The untrimmed whole western blots.

**Additional file 2.** Specific steps of using starBase v3.0.

## Data Availability

Not applicable.

## Abbreviations

ANOVA: Analysis of variance
ATCC: American type culture collection
BDNF-AS: Brain derived neurotrophic factor antisense RNA
cDNA: Complementary DNA
ceRNA: Competing endogenous RNA
DMSO: Dimethylsulfoxide
FBS: Fetal bovine serum
GAPDH: Glyceraldehyde-3-phosphate dehydrogenase
LINC00689: Long intergenic non-protein coding RNA 689
lncRNA: Long noncoding RNA
MALAT-1: Metastasis associated lung adenocarcinoma transcript 1
miRNA: microRNA
mRNA: Messenger RNA
MTT: Methyl thiazolyl tetrazolium
NC: Negative control
OD: Optical density
PBS: Phosphate-buffered saline
PCa: Prostate cancer
PVDF: Polyvinylidene fluoride
RIP: RNA immunoprecipitation
RISC: RNA-induced silencing complex
RT-qPCR: Real-time quantitative polymerase chain reaction
SD: Standard deviation
SDS-PAGE: Sodium dodecyl sulfate polyacrylamide gel electrophoresis
shRNA: Short-hairpin RNA
SOCS2-AS1: Suppressor of cytokine signaling 2 antisense RNA 1
SPSS: Statistic package for social science
UTR: Untranslated region
