# Supplementary figures and images for: LINC00689 promotes prostate cancer progression via regulating miR-496/CTNNB1 to activate Wnt pathway
Source: Cancer Cell Int. 2020 Jun 5;20:215. doi: 10.1186/s12935-020-01280-1 (PMC7275594; doi:10.1186/s12935-020-01280-1)

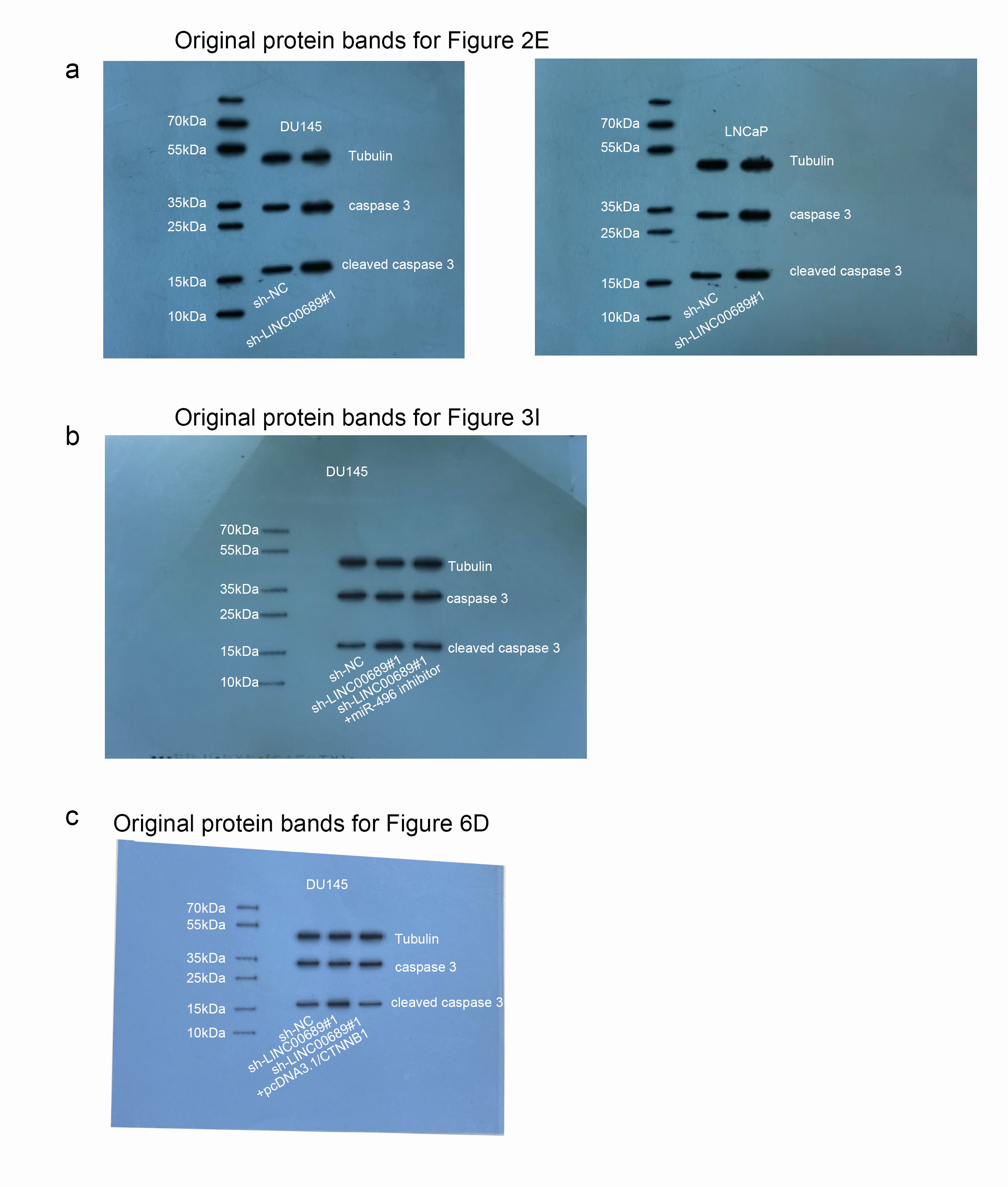

Supplement: Supplementary file 1 — Additional file 1. The untrimmed whole western blots. [file 12935_2020_1280_MOESM1_ESM.tif]

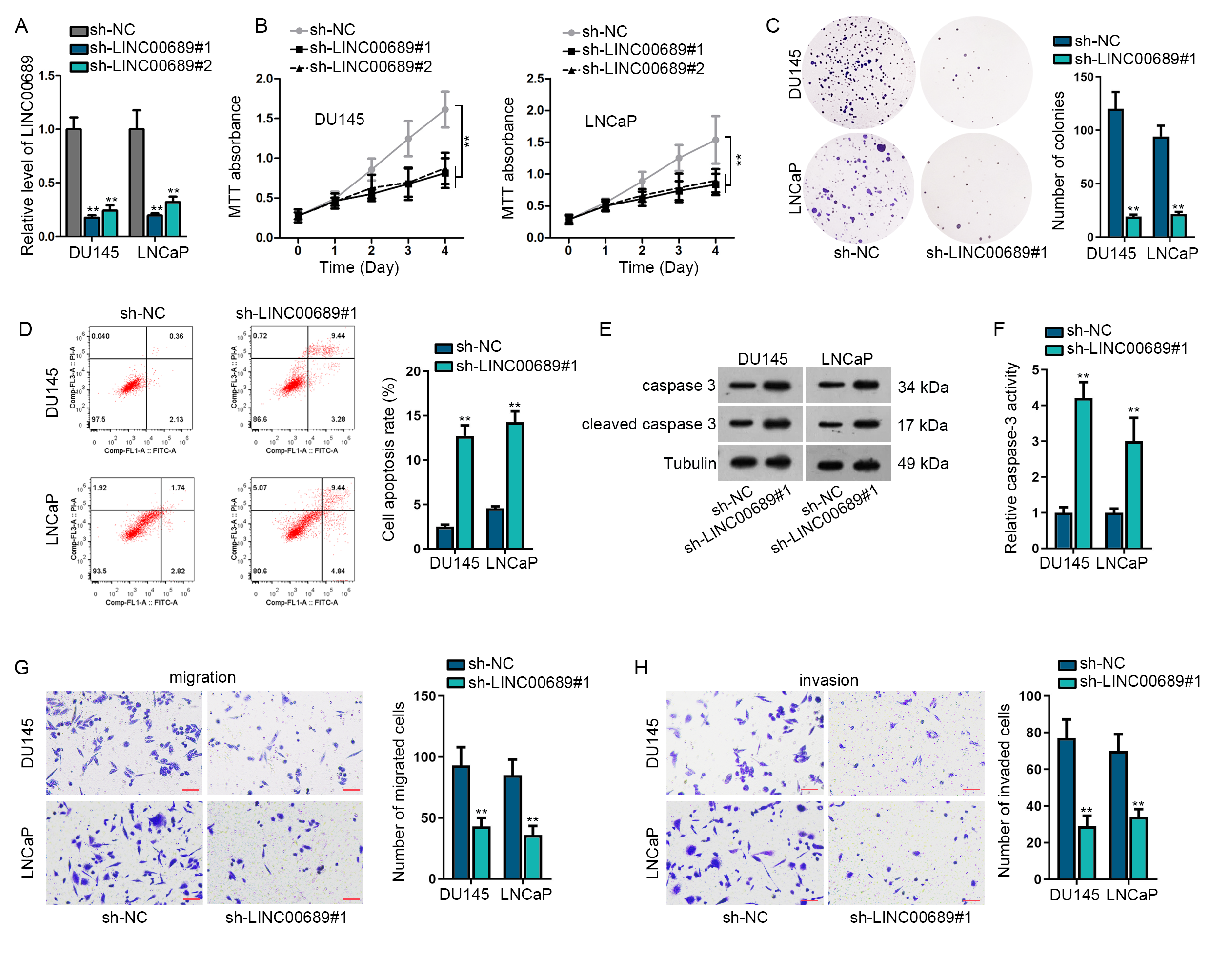

Supplement: Supplementary file 2 — Additional file 2. Specific steps of using starBase v3.0. [file 12935_2020_1280_MOESM2_ESM.tif]
